# Retraction Note: Simvastatin and Atorvastatin inhibit DNA replication licensing factor MCM7 and effectively suppress RB-deficient tumors growth

**DOI:** 10.1038/s41419-025-08275-8

**Published:** 2025-11-27

**Authors:** Juan Li, Jie Liu, Zheyong Liang, Fang He, Lu Yang, Pingping Li, Yina Jiang, Bo Wang, Can Zhou, Yaochun Wang, Yu Ren, Jin Yang, Jianmin Zhang, Zhijun Luo, Cyrus Vaziri, Peijun Liu

**Affiliations:** 1 Center for Translational Medicine, The First Affiliated Hospital of Xian Jiaotong University, Xi’an, 710061 Shaanxi China; 2 Key Laboratory for Tumor Precision Medicine of Shaanxi Province, The First Affiliated Hospital of Xian Jiaotong University, Xi’an, 710061 Shaanxi China; 3 Department of Pathology, The First Affiliated Hospital of Xian Jiaotong University, Xi’an, 710061 Shaanxi China; 4 Department of Breast Surgery, The First Affiliated Hospital of Xian Jiaotong University, Xi’an, 710061 Shaanxi China; 5 Department of Oncology, The First Affiliated Hospital of Xi’an Jiaotong University, Xi’an, 710061 Shaanxi China; 6 Department of Cancer Genetics, Roswell Park Cancer Institute, Buffalo, NY 14263 USA; 7 Department of Biochemistry, Boston University School of Medicine, Boston, 02118 MA USA; 8 Department of Pathology and Laboratory Medicine, University of North Carolina at Chapel Hill, Chapel Hill, NC USA

Retraction Note to: *Cell Death and Disease* 10.1038/cddis.2017.46, published online 16 March 2017

The Editors-in-Chief have retracted this article. Image integrity concerns were found within figure 1, between figures 1 and 2, between figures 2 and 3, and between figures 3 and 4. The images provided by the authors were not sufficient to resolve the concerns. The Editors have lost confidence in the data which supports the conclusions of the article.

Authors Juan Li and Peijun Liu disagree with this retraction. All other authors have not responded to the publisher regarding this retraction.

